# Peroxisome Proliferator-Activated Receptors (PPAR)γ Agonists as Master Modulators of Tumor Tissue

**DOI:** 10.3390/ijms19113540

**Published:** 2018-11-09

**Authors:** Daniel Heudobler, Michael Rechenmacher, Florian Lüke, Martin Vogelhuber, Tobias Pukrop, Wolfgang Herr, Lina Ghibelli, Christopher Gerner, Albrecht Reichle

**Affiliations:** 1 Department of Internal Medicine III, University Hospital Regensburg, Hematology and Oncology, 93042 Regensburg, Germany; daniel.heudobler@ukr.de (D.H.); michael.rechenmacher@kr.de (M.R.); florian.lueke@ukr.de (F.L.); martin.vogelhuber@ukr.de (M.V.); tobias.pukrop@ukr.de (T.P.); wolfgang.herr@ukr.de (W.H.); 2 Department Biology, Universita’ di Roma Tor Vergata, 00173 Rome, Italy; ghibelli@uniroma2.it; 3 Institut for Analytical Chemistry, Faculty Chemistry, University Vienna, A-1090 Vienna, Austria; christopher.gerner@univie.ac.at

**Keywords:** anakoinosis, communicative reprogramming, nuclear transcription factors, metronomic low-dose chemotherapy, glitazones, all-trans retinoic acid, COX-2 inhibitor, master modulators, undruggable targets, therapy pillar, peroxisome proliferator-activated receptors (PPARs), energy homeostasis, metabolic regulations, organ cross-talk, cancer and reprogramming of energy metabolism, systems biology

## Abstract

In most clinical trials, thiazolidinediones do not show any relevant anti-cancer activity when used as mono-therapy. Clinical inefficacy contrasts ambiguous pre-clinical data either favoring anti-tumor activity or tumor promotion. However, if thiazolidinediones are combined with additional regulatory active drugs, so-called ‘master modulators’ of tumors, i.e., transcriptional modulators, metronomic low-dose chemotherapy, epigenetically modifying agents, protein binding pro-anakoinotic drugs, such as COX-2 inhibitors, IMiDs, etc., the results indicate clinically relevant communicative reprogramming of tumor tissues, i.e., anakoinosis, meaning ‘communication’ in ancient Greek. The concerted activity of master modulators may multifaceted diversify palliative care or even induce continuous complete remission in refractory metastatic tumor disease and hematologic neoplasia by establishing novel communicative behavior of tumor tissue, the hosting organ, and organism. Re-modulation of gene expression, for example, the up-regulation of tumor suppressor genes, may recover differentiation, apoptosis competence, and leads to cancer control—in contrast to an immediate, ‘poisoning’ with maximal tolerable doses of targeted/cytotoxic therapies. The key for uncovering the therapeutic potential of Peroxisome proliferator-activated receptor γ (PPARγ) agonists is selecting the appropriate combination of master modulators for inducing anakoinosis: Now, anakoinosis is trend setting by establishing a novel therapeutic pillar while overcoming classic obstacles of targeted therapies, such as therapy resistance and (molecular-)genetic tumor heterogeneity.

## 1. Introduction

Peroxisome-proliferator-activated receptors (PPARs) line up in the group of nuclear receptors and encompass three receptors PPARα, PPARγ, and PPARδ, which concertedly and multifaceted have impact on regulating tumor growth [1]. From metabolic disease, the resolution of insulin resistance by PPARγ and combined PPARα/γ agonists, as well as long-term outcome in patients with type II diabetes, we learned a lot on simultaneous PPARα/γ stimulation. A specific PPARγ agonist has been withdrawn from the market, as rosiglitazone was associated with a significant increase in the risk of death from cardiovascular causes, from myocardial infarction [2]. The beneficial effects of the dual PPARα/γ agonist, pioglitazone, namely the reduction of mortality, including non-fatal myocardial infarction and stroke in patients with type 2 diabetes who are at high risk concerning macro-vascular events, shed light on the multi-level concerted activity profile of PPARα and PPARγ in diabetes [3]. Particularly, these clinical trials in patients with diabetes type II highlight the striking anti-inflammatory component of PPARα. The initial hypothesis that efficacious anti-inflammatory therapy may also control advanced cancer could be confirmed by introducing pioglitazone in treatment of refractory metastatic cancer [4,5]. From pre-clinical data, the appropriate PPARα agonist for cancer treatment has to be defined, yet [1].

Nuclear receptors (NRs) encompass a huge heterogeneous group of ligand-controlled transcription factors, endocrine, orphan and adopted receptors [6]. Schedules for cancer treatment using ligand-mediated modulation of NRs are well-established, particularly the blockade of endocrine NRs in prostate and breast cancer or the stimulation with high-dose glucocorticoids in lymphoma or multiple myeloma [7,8,9]. In contrast, adopted NR agonists only hesitantly found their way into cancer treatment, e.g., retinoid X receptor (RXR) and retinoic acid receptor (RAR) receptor agonists for treatment of T-cell lymphoma and promyelocytic leukemia, respectively [10,11].

A further NR agonist, pioglitazone, a dual peroxisome-proliferator-activated receptor (PPAR) α/γ agonist, is now starting to blaze the trail for therapy of metastatic tumor diseases and it will be discussed in more detail [12].

Therapeutically intended stimulation of adopted NRs for tumor control is in striking methodologic contrast to blocking endocrine NRs with antagonists or inducing direct cytotoxicity with high-dose glucocorticoids, opens a novel view on tumor pathophysiology, and finally, implies a change of treatment paradigms [13].

Following oncogenic events, dysregulated homeostatic pathways and transcription factors in tumor tissues are communication-technically accessible via endocrine, orphan, and adopted NR agonists or more general, via master modulators, a term summarizing regulatory active, less toxic drugs administered at regulatory active dose levels [12,14]. ‘Master modulators’ of tumors, i.e., transcriptional modulators, metronomic low-dose chemotherapy, epigenetically modifying agents, protein binding pro-anakoinotic drugs, such as COX-2 inhibitors, IMiDs, etc., are aiming at attenuation of cancer-associated hallmarks or at establishing novel biologic hallmarks linked to tumor control. ‘Master modulators’ deploy therapeutic activity via regulatory accessible structures, functions, and hubs in tumor tissue, thereby e.g., reestablishing differentiation and apoptosis competence (Table 1) [12].

NRs are involved in regulating multifold biologic processes in normal and tumor tissue [15,16,17]. Clinical trials have shown that ‘normalization’ of dysregulated transcription factors with NR agonists belongs to a pivotal, clinically relevant concept, and it finally constitutes a novel therapeutic pillar for treatment of (refractory) metastatic cancer [12]. However, in relation to the multitude of orphan and adopted nuclear receptors, the clinical impact of corresponding nuclear receptor agonists has not been nearly exploited in the clinical setting.

Multi-level activity profiles on single cell compartments, tissues, or the whole organism are characteristic for NR agonists [12]. Multifaceted clinically beneficial changes in tumor behavior are based on the ubiquitous availability of NRs in tissues. The distribution of single NRs, however, is tissue-specifically varying, implicating tissue-, and as shown, cancer-specific activity profiles [6,18]. Moreover, the kind of ligand, i.e., a synthetic or natural hormone and/or lipophilic drug, additionally, has major impact on multi-level outcome [19,20].

The ligand induced physical activity profile of NRs is dependent on multifold system-specific co-variables [21]. Their receptor and non-receptor mediated activity profile may explain and provide an insight in the multiplicity of biologic effects based on the predominantly regulatory and coordinating cell and tissue activities of NRs [22,23,24]. Therefore, interpretation and prediction of biologic outcome on the different observation levels within an organism is difficult and only accessible by application of novel technologies for monitoring ligand mediated biologic activities while treating metastatic tumors with nuclear receptor agonists.

Ligand induced structural changes of NRs facilitate binding at nuclear receptor response elements (NRREs) across the genome, but also the recruitment of co-regulators and interaction with other transcription factors, which may be again context-dependently activated or inhibited [25,26,27]. The obvious communication guided activity profile of NRs explicates why biologic read-outs may be contradictory depending on respective boundary conditions or systems stages, particularly in diseased organs [12].

When considering the context-dependent regulatory activity profile of NRs and the fact that cancer is constituted by complex dysregulation of transcription factors and homeostatic pathways, the following question arises: what kind of paradigms must be assumed for introducing NR agonists as attractive clinical targets, here, in particular, the α and γ variant of the peroxisome-proliferator-activated receptors (PPARs)? Secondly, may be the novel treatment approach, including agonists of nuclear receptors, universally applicable for treatment of metastatic, and refractory cancer and hematologic neoplasia?

## 2. Peroxisome Proliferator-Activated Receptor γ (PPARγ)/Cyclooxygenase-2 (COX-2) Expression in Tumors

Modulating COX-2 activity influences the local availability of PPAR ligands. Therefore, COX-2 indirectly modulates PPAR activity. Though acting on different signaling pathways, COX-2 and PPARγ modulate common molecular targets. Thus, COX-2 and PPARγ may concertedly inhibit cancer development [28]. Thereby, COX-2 inhibitors may act as partial PPARγ agonists [29], or PPARγ agonists as partial COX-2 inhibitors and suppressors of PGE2 synthesis [30,31].

Because of the close interaction of COX-2 and PPARγ, the differential expression in many human tumors, and the emerging possibilities to use them as targets for tumor therapy, we studied the correlation of PPARγ/COX-2 immunoreactivity with tissue microarrays (TMA) in a broad spectrum of histologic tumor types in comparison to normal tissue. In malignant melanoma, we focused on the correlation between clinic-pathologic features and outcome of patients with malignant melanoma (MM) [18].

TMA consisted of normal and tumor tissues (*n* = 3448) from 47 organs and tissue entities, including skin neoplasms (*n* = 323) of melanocytic (MM, benign nevi) and non-melanocytic origin (squamous cell carcinomas, basal cell carcinomas, Kaposi sarcomas, histiocytomas, capillary hemangiomas, sebaceous adenomas) [18].

COX-2 and PPARγ expression assays showed differential expression in almost every tissue type as well as in normal vs. neoplastic tissue: i.e., a continuous increase in COX2 expression from prostatic hyperplasia to prostatic intraepithelial neoplasia (PIN), to organ-confined prostate cancer, to castration-resistant prostate cancer, and to metastatic disease. In contrast, PPARγ expression decreases from the organ confined to the metastatic stage and increases again to the castration-resistant stage.

It could not be confirmed that COX-2 and PPARγ are inversely expressed in the human breast cancers, as breast cancer histologies are quite heterogeneous and differentially express COX-2 and PPARγ [32]. Activation of PPARγ may cause COX-2 inhibition or the down-regulation of COX-2 expression [33], whereas the inhibition of COX-2 resulted in PPARγ activation [34] or up-regulation of PPARγ expression [35].

Additional series of TMAs consisted of 88 MM with follow-up data, 101 MM metastases, and 161 benign nevi. A further TMA (*n* = 194) consisted of MM metastases from 36 patients with metastatic stage IV melanoma who had participated in a randomized phase II trial using a stroma-directed biomodulatory approach combining COX-2/ PPARγ-targeting with metronomic low-dose chemotherapy [18].

COX-2 and PPARγ immunoreactivity were paralleled and significantly increased from benign nevi (51%/0%) to primary MM (86%/22%) and MM metastases (91%/33%; *p* < 0.001, respectively). In the case of primary MM, positive COX-2 staining was associated with advanced Clark levels (*p* = 0.004) and shorter recurrence free survival (*p* = 0.03). However, PPARγ expression in primary MM was not associated with any of the clinic-pathologic characteristics or tumor progression and overall survival [18].

On the other hand, patients (*n* = 36) with PPARγ positive MM metastases who had been treated either with pro-anakoinotic metronomic low-dose chemotherapy (trofosfamide) alone or combined with COX-2/ PPARγ -targeting drugs, i.e., rofecoxib and pioglitazone, showed a significant advantage concerning progression-free survival (*p* = 0.044), but not overall survival (*p* = 0.179). Expression of COX-2 (score 2+–3+) in the metastases, however, was not associated with overall and progression-free survival, respectively [36].

We conclude that the expression of COX-2 and PPARγ is a frequent finding in the progression of MM. Regarding primary MM, the expression of COX-2 indicates an increased risk of tumor recurrence, i.e., melanoma progression.

In metastatic MM, the expression of PPARγ may serve as positive predictive marker of potential responsiveness to anakoinosis-inducing stroma-targeted therapy [36].

## 3. PPARγ Expression in Tumor Stroma

Apart from specifically stroma cells targeting drugs, some well-established pro-anakoinotic drugs, among them NR agonists, have revealed antitumor activity by unfolding pleiotropic biological effects. In this context thiazolidinedione derivatives such as pioglitazone are of special interest as they exert both a direct anti-tumor and a broad spectrum of stromal activities, including modulation of immune response, angiogenesis, and inflammation [37].

Stroma cell-specific NR signatures have to be suggested to collectively influencing tumor proliferation and metastasis [38]. Compartment specific NR expression and their context-dependent interaction with coregulators of NRs facilitate a complex dysregulated communicative network of transcription factors supporting multifold biologic hallmarks and tumor growth. On this presumably stage- and tumor-dependent background of NR expression, the profiling of NRs in stroma cells is urgently warranted for providing further rationales for combined transcriptional modulation in a therapeutic setting.

## 4. Induction of Anakoinosis with Master Modulators 

Expression patterns of PPARγ in histologic different tumor tissues, both in tumor cells and adjacent stroma cells indicate histology and even tumor stage specific characteristic patterns, even, as shown, with predictive impact. Tumor-specific patterns of PPARγ expression support that PPARγ is strongly involved in maintaining homeostatic processes by adapting lipid and carbohydrate metabolism to respective tumor specific conditions, and by controlling tumor suppressor gene expression for keeping homeostatic pathways under tumor growth-promoting conditions, such as Wnt, Hippo-YAP pathway, etc. [27,39,40,41].

Consecutively, many experimental data indicate that PPARγ agonists may modulate multifold biologic hallmarks in cancer: Cell cycle, differentiation, proliferation, apoptosis, and oxidative stress, innate immunity, angiogenesis, and inflammation [42,43,44].

However, in most trials, thiazolidinediones (TZD) do not show any clinically relevant anti-cancer activity when used in mono-therapy (Table 2). Therefore, clinical inefficacy contrasts ambiguous pre-clinical data mostly favoring anti-tumor activity, but also tumor promotion. Thus, most review papers come to no consistent conclusion about the clinical use of PPARγ agonists for cancer treatment.

In contrast, if thiazolidinediones are combined with additional regulatory active drugs, so-called ‘master modulators’ of tumors, i.e., transcriptional modulators, metronomic low-dose chemotherapy, epigenetically modifying agents, protein binding pro-anakoinotic drugs, such as COX-2 inhibitors, IMiDs etc., clinical results indicate the relevant communicative reprogramming of tumor tissues, i.e., anakoinosis, meaning ‘communication’ in ancient Greek (Table 1).

## 5. Keys for Uncovering the Therapeutic Potential of PPARγ Agonists: Selecting the Appropriate, Histology-Independent Combination of Master Modulators

Clinical data reveals that most regulatory active drugs, i.e., master modulators of tumor tissues, exert only a modest or no monoactivity in cancer treatment (Table 2). Also, metronomic low-dose chemotherapy has just modest activity in randomized comparisons [69,70,71,72,73,74,75].

However, combining master modulators in 17 different histologic tumor entities leads to impressive, and, interestingly, highly diversified tumor responses up to continuous complete remission (Table 2). Moreover, single combinatory schedules of master modulators, including pioglitazone, are cross-responsive among quite different tumor histologies [12,51]. Cross-responsiveness now clearly indicates that different tumor histologies share identical patterns of hallmarks of cancer and constitute similar physical organizations of hallmarks, so called rationalizations of hallmarks, despite underlying (molecular-) genetic tumor heterogeneity (Table 1).

**Figure 1:**
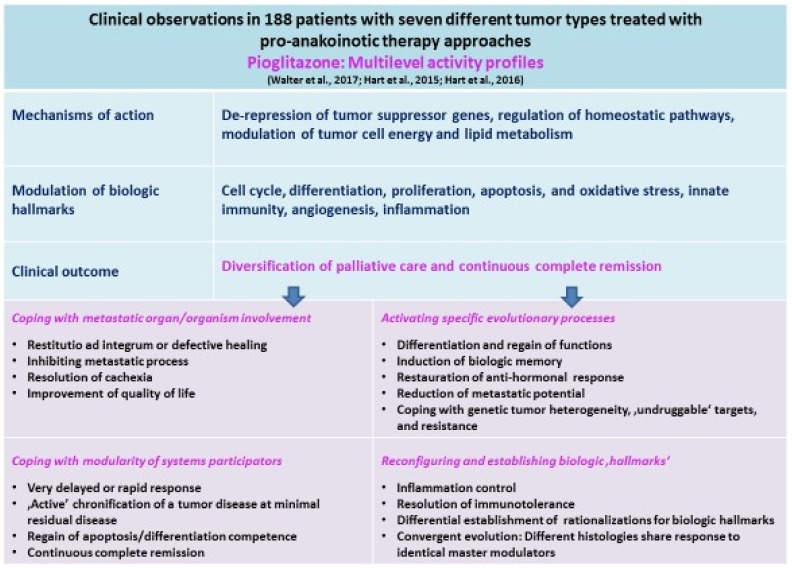
Pioglitazone in tumor therapy regulates the communicative interface of transcriptional modulation, lipid and carbohydrate metabolism, particularly in combination with additional master modulators. Thus, tumor-promoting pathways can be functionally attenuated without direct blocking tumor-promoting pathways or by shutting off tumor-associated cellular compartments. Clinical equivalents are diversification of palliative care, even continuous complete remission.

Thus, the clinically used top-down approaches reveal that tumor phenotypes are not dominantly minted and are associated with multifold recessively developing tumor features, which may be accessible for the concerted activity of regulatory active drugs. The specific therapeutic and clinically relevant access to tumor systems prompted us to choose for the procedure the term ‘anakoinosis’, communicative reprogramming [12]. The term anakoinosis reflects how regulatory active drugs may concertedly induce major tumor response, obviously by altering validity, i.e., availability on demand at distinct time points, and denotation, i.e., current functional impact at a distinct systems stage of tumor-promoting pathways (Table 1, Figure 1).

The fact that communication rules may change validity and denotation of systems objects may be generally attributed to communication.

The successful concerted administration of pro-anakoinotic drug combinations, including PPARγ agonists in the clinical setting, may now explain multiple, from the clinical point of view cumulatively vague, as always context-dependent and often opposing results on the function of PPARγ agonists [1,6,24,76,77,78,79,80,81,82,83,84]. The missing conception for integrating pre-clinical results in clinical practice underlines missing communication-based therapeutic paradigms provided by an evolution-adjusted tumor pathophysiology and implies an unjustified hesitant introduction of master modulators, including PPARα/γ agonists, like pioglitazone in tumor therapy (Table 1).

Pre-clinically synergistic activities of PPARγ have been reviewed, particularly combinations with chemotherapy, besides RXR ligands and statins [82].

### 5.1. Poor Monoactivity of PPARγ Agonists Across Different Tumor Histologies

Monoactivity of glitazones in cancer patients is very modest, whereas strong activity is well established in single tumor histologies for dexamethasone, all-trans retinoic acid, and bexarotene [11,85].

Metabolically active drugs, such as metformin or PPARγ/α agonists, are considered as chemopreventive agents [86,87]. Metformin may prolong survival in cancer patients following surgery, but only in distinct histologic tumor types, as retrospective studies are indicating [88]. Nevertheless, very recent data shows a mechanistic link between glucose metabolism and cancer being mediated by TET2-function [89].

Agonists of ‘adopted’ orphan receptors commonly have poor monoactivity in interventional cancer trials [59,90], in contrast to hormones and cytokines [91,92]. Particularly, dexamethasone plays a decisive role in the induction treatment for acute lymphocytic leukemia or multiple myeloma [90].

### 5.2. PPARγ Agonists in Pro-Anakoinotic Combination Therapy with Master Modulators

Stromal cells or normal epithelial cells are not equipped for directly sensing tumor promoting genetic or molecular-genetic aberrations in neighboring malignant transformed cells. Adjacent non-tumor cells, however, sense dysregulations in homeostatic pathways. Thus, it is not surprising that hair follicle epithelia may spontaneously eliminate malignant transformed counterparts, irrespective of the underlying oncogenic events by sensoring dysregulated homeostasis [93].

Therefore, our commonly used therapeutic procedure, based on ‘sensing’ and consecutively blocking oncogenic pathways, is completely different from the pathophysiological based in vivo recognition of equivalences of malignancy by non-tumor cells, i.e., dysregulated homeostatic processes. All tumor-associated (molecular-) genetic aberrations are profoundly involved in dysregulations of homeostatic pathways [14]. Thus, tumors can be considered as a big dysregulated network of transcription factors. Just the communicatively evolving transcriptional system irregularities may be recognized as therapeutic target for master modulators. Master modulators are equipped with the capacity for ‘normalizing’ homeostatic networks on quite different topographic levels: the tumor’s different cell compartments, the tumor and the tumor-harboring organ, and finally, the tumor and the whole organism (Table 1) [12].

Dysregulated homeostatic pathways represent, even if complex for pre-clinical evaluation, a pivotal therapeutic tool for ‘normalizing’ dysregulated homeostatic processes via master modulators, including agonists of nuclear transcription factors. NR antagonists are well integrated in clinical use and are here excluded from consideration. The review, particularly, concentrates on pioglitazone, a dual receptor agonist for PPARα/γ.

As shown to some extent, tumor-associated transcriptional dysregulation provides access for specific pro-anakoinotic effects via master modulators, including NR agonists. Moreover, histologically different tumor types share distinct communication-derived dysregulations, independent of the oncogenic background and show cross-reactivity to distinct systems adapted combinations of master modulators [12].

Cancer-specific impressive transcriptional dysregulation in comparison to the homeostatically well-balanced repertoire of transcription factors in normal organ tissue might be responsible for the modest toxicity profile of therapies, including combinations of master modulators. Therapeutic effects of combinations of master modulators should be to some degree neglectable in homeostatically well balanced, normal tissues, as they do not lay themselves open to therapeutic attack with master modulators selected for special evolution-related operative conditions in tumor tissue (Table 1) [12].

**Figure 2:**
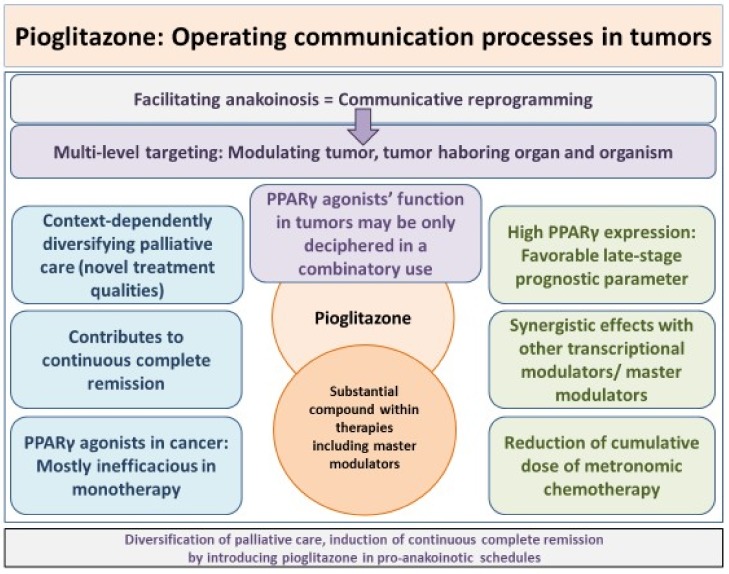
Pioglitazone, operating communication processes in tumors: Clinical relevance.

The top-down approach only has established how agonists of nuclear transcription factors, or generally master modulators, might communicatively interact for diversifying palliative care or even for inducing continuous complete remission. Additionally, maximal tolerable doses can be yield up, as pro-anakoinotic acting, lower doses are sufficient for achieving a therapeutically relevant response (Figure 2).

#### 5.2.1. PPARγ Agonists Combined with Metronomic Low-Dose Chemotherapy/Demethylating Agents

Metronomic low-dose chemotherapy is still not established in routine therapy of neoplasia, as randomized comparisons often show no advantage for metronomically scheduled chemotherapy [69,70,71,72,73,74,75]. However, pre-clinical and clinical data give hints that the addition of classic targeted therapies or master modulators may improve outcome, even may diversify palliative care, and may contribute to continuous complete remission [94].

There are several reasons to include metronomic low-dose chemotherapy in the group of master modulators of tumor tissues. By adding pioglitazone and a COX-2 inhibitor, or an additional transcriptional regulator, such as a glucocorticoid, all-trans retinoic acid or interferon-α, outcome in refractory metastatic tumor disease could be improved up to continuous complete remission (Table 2). Additionally, chemotherapy doses could be reduced up to a quarter or third of the respective cumulative dose, which would be administered as pulsed therapy every three to four weeks, without loss of efficacy. Therefore, currently the question remains unanswered, which is the lowest, still regulatory active dose of metronomic chemotherapy when combining several master modulators [12].

In metastatic melanoma, the addition of pioglitazone to metronomic low-dose chemotherapy and COX-2 inhibitor has important therapeutic impact on outcome, as indicated in the paragraph ‘PPARγ agonist plus COX-2 inhibitor’.

An important link between pioglitazone and metronomic chemotherapy may be physically explained. Pioglitazone sensitizes metronomic low-dose chemotherapy response by up-regulation of both, the receptor for the angiogenesis inhibitor thrombospondin 1, CD 36, and the phosphatase and tensin homolog PTEN [95,96,97,98].

#### 5.2.2. PPARγ Agonists Plus Dexamethasone

Interacting with transcription factors as well as other cell-signaling systems nuclear receptors are important regulators in innate and adaptive immunity. PPARs, LXRs, and the glucocorticoid receptor (GR) may act together and thereby integrate local and systemic responses to inflammation by p65/IRF3-independent mechanisms [99]. Cooperating with the GR PPARs und LXRs synergistically transrepress distinct subsets of toll-like receptor-responsive genes. Thus, the combinatorial control of homeostasis and immune responses by nuclear receptors may specify the response and suggest novel approaches for treatment of pro-inflammatory tumor diseases [99].

In a series of quite different tumor histologies, the cross-responsiveness to dual transcriptional modulation with pioglitazone and glucocorticoid could be nicely shown, when added to metronomic low-dose chemotherapy. The concept has been tested in multiple myeloma, Hodgkin disease, and Langerhans cell histiocytosis, all inflammation-triggered diseases. C-reactive protein control in peripheral blood was indicative for response [12,63].

Preclinical data show that thiazolidinediones induce growth arrest and apoptosis of Waldenström’s macroglobulinemia cells, at concentrations that are relevant to those achieved in previous clinical uses of these drugs [100].

From pre-clinical data on prostate cancer, PPARγ agonists may be acting, in part, by inhibiting transactivation of androgen-responsive genes [101]: Peroxisome proliferator-activated receptor γ agonists may down-regulate prostate-specific antigen expression in human prostate cancer [102].

Positive correlation between PPARγ and fatty acid synthase (FASN) protein in prostate cancer cell lines and synergism between TZDs and FASN blockers could be shown in prostate cancer cell viability reduction and apoptosis induction. [103].

Androgen receptor and Wnt/β-catenin/Tcf are cross-regulated. RAR/RXR, GR, thyroid receptor (TR), vitamin D receptor (VDR), estrogen receptor (ER), and PPAR modulate canonical Wnt signaling in dynamic manner with striking cell line- and tissue-specific differences indicating selective therapeutic access and requiring deciphering for combined transcriptional modulation in a therapeutic setting [40]. This fact may give hints for the combinatorial use of receptor agonists and antagonists.

Dual transcriptional modulation with glucocorticoids and pioglitazone in combination with metronomic low-dose chemotherapy and COX-2 inhibitor improved in a historic comparison overall survival in high-risk patients with castration-resistant prostate cancer from 19 months to more than three years. The addition of imatinib had no impact in this trial [56].

Thus, rapidly progressive castration-resistant prostate cancer responded to the same therapy principle as refractory Hodgkin disease, multiple myeloma, and Langerhans cell histiocytosis, but the communication-technically provided dysregulated systems targets seem to be different. Castration-resistant prostate cancer is only in rare cases that are associated with pro-inflammatory systems reaction, and C-reactive response in serum was no indicator for response as in refractory Hodgkin disease, multiple myeloma, and Langerhans cell histiocytosis [54,63,64,65]

#### 5.2.3. PPARγ Agonists Plus All-Trans Retinoic Acid

The combination of azacitidine plus all-trans retinoic acid and pioglitazone may induce ex vivo granulocytic differentiation in more of 50% of blasts from acute myelocytic leukemia [67]. Moreover, these granulocytes regain phagocytic activity, when exposed to *E. coli* (unpublished data). Clinically, it is possible to induce continuous complete remission in acute myelocytic leukemia with the triple combination, while using only about 50% of the recommended dose of azacitidine [67,104].

A randomized trial in refractory acute myeloid leukemia (AML) is on-going, comparing the approved dose of azacitidine in comparison to the dose per square meter plus all-trans retinoic acid and pioglitazone.

Synergistic activity of dual transcriptional modulation has been well established in pre-clinical studies, for example, for pioglitazone and all-trans retinoic acid in tumor cell lines of different histology [68,105,106,107,108], but also for glitazones in combination with chemotherapy [76]. Clinical trial designs translated these pre-clinical results comparatively hesitantly.

#### 5.2.4. PPARγ Agonists Plus Interferon-α

In renal clear cell carcinoma (RCC), IL-6 is a prognostic factor for survival [109]. Vice versa, in anakoinosis-inducing trials, including pioglitazone, C-reactive protein response to anakoinosis-inducing therapy is indicating tumor response [110].

Interferon-α is an approved drug in RCC and acts strongly anti-inflammatory by inducing circulating tumor necrosis factor receptor p55 and mediates a rapid and strong C-reactive protein (CRP) decrease by inhibiting TNFα. RCC is a tumor, producing directly CRP, not only mediated via liver [111].

In a first trial, pioglitazone combined with metronomic chemotherapy and COX-2 inhibitor relatively poor response, mainly stable disease could be observed in > third line situation. The addition of low-dose interferon-α opened the possibility to induce histologically proven remission in resistant metastatic RCC, which translated in continuous complete remission, now lasting > 10 years in single patients [5].

Interestingly, interferon-α is active in renal cell carcinoma, both in combination with retinoids or pioglitazone [5,112,113].

#### 5.2.5. PPARγ Agonists Plus COX-2 Inhibitor

COX-2 inhibition is tightly regulating cellular levels of fatty acids and their derivatives, which are mainly derived from the lipoxygenase and cyclooxygenase pathways. Modulating COX-2 activity influences the local availability of PPAR ligands, therefore indirectly PPAR activity [114].

Inhibiting the canonical Wnt signaling pathway, nonsteroidal anti-inflammatory drugs as well as PPARγ agonists are candidate agents for chemoprevention. Celecoxib suppresses cancer stemness and the progression of hepatocellular carcinoma via activation of PPARγ and up-regulation of PTEN [115]. COX-2 and peroxisome proliferator-activated receptor delta are involved in important growth promoting signaling pathways in human hepatocellular carcinoma [116]. The non-steroidal anti-inflammatory drugs (NSAID)-dependent inhibiton of COX-2 and activation of PPARγ has been shown to suppress cancer stem cells in colon cancer [97]. Celecoxib, for instance, induces up-regulation of PTEN in N1-S1 cells. This process can be enhanced by rosiglitazone. Moreover, it has also been shown that celecoxib increases PPARγ expression and PTEN activity in wild-type and COX-2-deleted Huh7 cells [117]. Concerning the mechanism, within the PTEN promoter, two putative PPARγ binding sites have been identified [96].

Anti-tumor-effects of a cyclooxygenase-2 inhibitor and a peroxisome proliferator-activated receptor γ agonist have been also demonstrated in an in vivo mouse model of spontaneous breast cancer [43,118].

In a series of clinical trials, we used pioglitazone combined with rofecoxib or etoricoxib. From one randomized trial in metastatic melanoma, at least the impact of pioglitazone in addition to COX-2 inhibitor and metronomic low-dose chemotherapy may be delineated. High PPARγ expression in melanoma cells is a favorable prognostic factor for progression-free survival. PPARγ is a late stage predictive marker in metastatic melanoma, and PFS is significantly improved by adding pioglitazone to a pro-anakoinotic schedule, including metronomic low-dose chemotherapy and COX-2 inhibitor (Table 2).

It is not possible to directly estimate the clinical impact of the COX-2 inhibitor from single arm pioglitazone and COX-2 inhibitor, including trials in addition to metronomic low-dose chemotherapy.

#### 5.2.6. PPARγ Agonists and IMiDs

In an animal model, pomalidomide enhances the expression of PPARγ and CCAAT/enhancer binding protein α (C/EBPα), as well as the activity of lipoprotein lipase (LPL) and fatty acid synthetase (FAS). The pro-inflammatory activity of TNFα has the opposite effect on the biochemical indexes and genes that are related to lipid deposition in the liver [119].

Additional experimental data on tumor growth inhibition implicate thalidomide as being involved in the PPARγ pathway. Thalidomide and pomalidomide increase PPARγ protein dose-dependently, also activity of peroxisome proliferator response element [120].

In a clinical trial on multiple myeloma, we successfully used pioglitazone and lenalidomide plus low-dose metronomic chemotherapy and glucocorticoid for rescuing patients following failure of lenalidomide containing regimens in > third line therapy. All of these modulating activities justify for including IMiDs to master modulators of tumor tissue [63].

### 5.3. PPARγ Agonists in Pro-Anakoinotic Combination Therapy Combined with Targeted Therapy

#### 5.3.1. Pioglitazone and Imatinib

Pioglitazone with imatinib in CML may reduce minimal residual disease. PPARγ agonists target chronic myeloid leukemia (CML) quiescent stem cells in vitro by decreasing transcription of STAT5. A fact that was also shown in multiple myeloma for STAT3. A phase III trial is on-going in France when comparing imatinib versus imatinib plus pioglitazone, as front-line therapy for CML [121].

The addition of imatinib in prostate cancer had no impact on outcome, although there are strong pre-clinical results indicating an impact of imatinib on potentially clinical relevant PDGFR inhibition in prostate cancer [57].

#### 5.3.2. PPARγ and Mechanistic Target of Rapamycin (mTOR) Inhibitor

An additive or synergistic activity of thiazolidinediones and mTOR inhibitors can be suggested from pre-clinical data. Activation of PPARγ by thiazolidinediones leads to inhibition of cell growth and proliferation via key pathways of the Insulin/IGF axis, such as PI3K/mTOR, mitogen-activated protein kinase (MAPK), and GSK3-β/Wnt/β-catenin cascades. This signal pathways regulate cancer cell survival, cell reprogramming, and differentiation [84]. The inhibitory effect of rosiglitazone on non-small cell lung cancer (NSCLC) cell growth was enhanced by the mTOR inhibitor rapamycin. Rosiglitazone, via up-regulation of the PTEN/AMPK and down-regulation of the Akt/mTOR/p70S6K signal cascades, inhibits NSCLC cell proliferation through PPARγ-dependent and PPARγ-independent signals [122].

In refractory Hodgkin disease and MM, an mTOR inhibitor was introduced in addition to pioglitazone, metronomic low-dose chemotherapy, and COX-2 inhibitor; in Hodgkin lymphoma, a glucocorticoid was used, additionally. Metastatic uveal melanomas responded with long-term disease stabilization, improvement of Eastern Cooperative Oncology Group (ECOG) status and resolution of cachexia. In fourth-line PET negative complete remissions were achieved in Hodgkin lymphomas. All patients received allogeneic blood stem cell transplantation in first complete remission [51,66].

Both neoplasia are poorly responding to mTOR inhibitors, only. Thus, also the use of classic targeted therapy, here, the mTOR inhibitor may be repurposed [123].

## 6. Specific Methodological Aspects of Anakoinosis Inducing Therapies

### 6.1. Communication Tools

The successful use of pro-anakoinotic therapy approaches gives hints that generally available evolutionary strategies of single cells and tissues may be therapeutically recalled and accessed, particularly in the diseased stage, characterized by transcriptional dysfunctions.

As clinically shown, operating communication tools, including master modulators, evolves therapeutic capacity for biologically ‘neutralizing’ tumor promoting systems features without blocking tumor-relevant pathways or without targeted elimination of cell compartments of the tumor. The suggestion of communication tools seems to oppose molecular-biologic thinking in networking pathways supposing structures, functions, and hubs with simplistically presumed invariant validity and denotation. However, those classic pathway paradigms disregard that each systems object, also that in a tumor, whatever it will represent physically, a structure, such as a molecule or cell, a function or hub, may be intrinsically or extrinsically, namely therapeutically, nudged by communication derived impulses for context-dependently changing its validity and denotation. Secondly, the identity of structures, functions, or hubs is always communicatively mediated, and necessarily includes and depends on the environmental conditions, functioning as boundary conditions, and integrates the scientific point of view, which is invariably subjected, even if it can be commonly objectively backtraced.

Communication within biologic systems works with the implicit understanding that (1) validity and denotation of systems objects, molecules, cells etc., is always context-dependent, (2) and may be therapeutically redeemed by master modulators via systems-immanent communication tools, which are determined by descriptively accessible communicative systems textures, including inter-systemic exchange processes. The difference between theory, the activity profile of systems participators under invariant ‘standard conditions’, and practice, the situative evolution-adjusted activity profile, may be bridged by operating communication tools inducing evolutionarily conserved and therapeutically retrievable evolutionary processes (Table 1, Figure 1) [124].

With the introduced paradigmatic changes, the circle can be closed, between multifaceted and contradictory pre-clinical results on the action of PPARγ agonists and unambiguous, reproducible clinical observations resulting from the combined use of master modulators, including NR agonists.

The clinical observations on therapies with master modulators also support experimental data that tumor development and progression is not only a matter of oncogenic events, but of the disease stage, an observation that is also supported by PPARγ expression and predictivity for progression-free survival in metastatic melanoma (Figure 2) [18].

NR agonists develop context and ligand dependent activity profiles. Therapeutic top-down approaches for treating refractory metastatic tumors and hematologic neoplasia indicate that the PPARγ agonists’ clinical function may be only deciphered in a combinatory use. Only by introducing several master modulators in therapeutic schedules, including, for example, PPARγ agonists, master modulators develop the capacity for mutually specifying and enhancing response, now up to a clinically relevant level, which can be hardly achieved with mono-therapy, as shown by the missing monoactivity of PPARγ agonists in cancer treatment [12].

Clinical read-outs following combined administration of master modulators are also multifaceted, but reproducible, and they are resulting in diversified, clinically meaningful, palliative care, or response may even disembogue in continuous complete remission. Situative and stage-dependently varying communication features on the respective topographic levels, tumor tissue, tumor-harboring organ, and organism represent the therapeutic counterpart to the diversified context-dependent pre-clinical observations (Figure 1). In case of cachexia, cachexia may be resolved in metastatic melanoma with PPARγ agonist, including schedules with master modulators [51,125].

Importantly, the combined activity profile of PPARγ agonists plus further master modulators is highly specific. However, tumors may either share the communicative systems contexts and therefore, also the therapeutic accessibility towards distinct combinations of master modulators, or may be in the worst case unresponsive, due to the presence of alternative communicative systems contexts, or alternative constitutions of identical hallmarks of cancer.

At this step, individualization of pro-anakoinotic therapy could take place by describing the evolution-adjusted tumor pathophysiology, for example, via serum proteomics and metabolomics [125,126].

### 6.2. What Is the Appropriate Model System: From Histology to ‘Evolution-Adjusted’ Tumor Pathophysiology?

The key for uncovering the therapeutic potential of PPARγ agonists is selecting the appropriate combination(s) of master modulators for inducing anakoinosis: Now, anakoinosis is trend setting by establishing a novel therapeutic pillar while overcoming classic obstacles of targeted therapies, such as therapy resistance and (molecular-)genetic tumor heterogeneity.

The clue is that different histologic tumor types share response to distinct combinations of master modulators. That means histologic systematics, in any case reaching its operational limitations in clinics, even by including molecular-pathology and molecular genetics, may be newly unlocked and re-systematized. For this purpose, the systematic specification of tumor-specific communication networks may be adducted, based on the ubiquitously available communication tools, and the evaluation of diversified rationalizations, i.e., physical constitutions of biologic hallmarks, including the hallmarks of cancer. Diversified rationalizations may constitute identical normative notions, for example, rapidly displacing growth of acute leukemias in bone marrow (Table 1). Thus, profound systematics of tumor-specific communication routes, not to be mixed up with the context-independently discussed tumor-promoting pathways, and knowledge about the situative physical constitution of rationalizations results in an ‘evolution-adjusted’ tumor pathophysiology, which may be prerequisite for the targeted selection of combinations with master modulators (Table 1). By operating anakoinosis in tumors, therapy may cope with the situative relativity of biologic systems, i.e., situative validity and denotation of systems objects in biologic systems. Master modulators may be successfully therapeutically applied for exploiting the possibilities of palliative care and for inducing continuous complete remission.

All communication guiding, validity, and denotation modulating structural, functional tools, including tuning of hubs, are principally therapeutically accessible with pro-anakoinotic drug cocktails, as shown for multiple histologic quite different tumor entities.

### 6.3. What Is the Appropriate Dosage of Pro-Anakoinotic Therapy?

Single dosages of master modulators, so the postulate, must sufficiently equip the tumor system with reprogramming capacity for attenuating tumor growth. The appropriate regulatory active dosage cannot be pharmacokinetically defined, yet. As clinically indicated, the combination of PPARγ agonists with metronomic chemotherapy facilitates dose-reduction of the cumulative chemotherapy dosage to a quarter or third of the pulsed dose given every three to four weeks without the loss of clinical efficacy.

The two different dose levels of pioglitazone 60 or 45 mg daily seem not to have any impact on response. In addition, patients with both, reduced doses of metronomic chemotherapy, and with dose-reduction of pioglitazone achieved significant clinical response [12].

Within the combined pro-anakoinotic therapy schedules, typical, but modest side effects, can be attributed to the administration of pioglitazone [57,58]. Peripheral edema Grade I to II occurred in 52.4 to 58.5%, including few Grade III toxicities in hepatocellular carcinoma due to pre-existing liver disease. Renal failure Grade I to II was observed in 13.2% of patients with hepatocellular carcinoma. Adverse events leading to dose adjustment or temporary interruption of therapy in the prostate cancer trial occurred in 13.8%, permanent discontinuation in 1.2%. In hepatocellular carcinoma, dose adjustments of pioglitazone (starting dose 60 mg daily) were performed in 33% of patients, no permanent discontinuation.

### 6.4. Pro-Anakoinotic Therapy Schedules: Indications and Diagnostics

Up-to-now, anakoinosis inducing therapies, including pioglitazone, have been administered in metastatic and refractory cancer and hematologic malignancies. From the results of these trials (Table 2) a proof of principle can be delineated, namely activity of anakoinosis inducing therapies in poor-risk patient populations. First steps in the direction of combining classic targeted therapy with anakoinosis-inducing schedules were successful (Table 2). When considering tumor response as a timely multi-step biologic approach, during those reprogramming and classic targeted steps are repetitively and/or simultaneously necessary for inducing long-term tumor response, differential valuable clinical endpoints may be biologically accessible, such as induction of biologic memory, ‘active’ long-term chronification of tumor disease, or, in the best case, continuous complete remission. Anakoinosis inducing therapies could be perspectival integrated in ideal manner in classic targeted approaches. Classic targeted therapies may be even repurposed with many possible implications for additional clinical approvals [123].

Monitoring of such anakoinosis-inducing therapies must be completely reorganized in comparison to targeted therapies, where the availability of the target on the tumor or stroma cell is used as indicator for possible tumor response. Now, multiple parameter analysis derived from proteome and metabolome analytics from serum or plasma might be helpful, before and during therapy.

## 7. Conclusions

A long way of failures accompanied the introduction of PPARγ agonists in tumor therapy. In contrast to NRs that are activated by hormones and the prompt incipient activity of hormones, adopted NRs have an intrinsic tissue- and stage-dependent pro-anakoinotic activity profile, which is pointed in tumor tissues with their severe dysregulation of transcription factors. However, PPARγ agonists are clinically irrelevant, as far as, for example, PPARγ agonists are used in mono-therapy. With respect to pioglitazone, the tumor systems related activity profile may be exclusively focused and up graded to a clinically meaningful range by introducing additional NR agonists, as shown for glucocorticoids, all-trans retinoic acid, or the transcriptional modulator interferon-α, or more generally, by adding master modulators. Under conditions of concerted activity of master modulators, it should be generally possible to elaborate and adopt combinations of master modulators inducing response in metastatic, refractory neoplasia, irrespective of the histologic origin.

Thus, with induction of anakoinosis, a novel therapy pillar may be introduced providing several advantages compared to classic targeted therapies:

Anakoinotic processes may cope with fundamental obstacles of classic targeted therapies, with tumor heterogeneity and poor risk parameters, with context-dependent validity and denotation of tumor-promoting aberrations and targets, with drug resistance or undruggable targets by targeting dynamic evolutionary processes, for example, multifaceted biologic steps that are necessary for establishing ‘active’ long-term tumor control or continuous complete remission [12]. Pro-anakoinotic therapies may inhibit further metastatic progression in case of metastatic disease (Table 1, Figure 1) [127].

Auspicious ‘personalized’ tumor therapy is now supplemented by a novel treatment methodology, which is at its beginnings but multifaceted adaptable to tumor systems stages. Importantly, metastatic tumors of quite different histologic origin may share communication features and may be reprogrammed with identical combinations of master modulators operating communication tools. Thus, in future, an evolution-adjusted tumor pathophysiology could be the driving force for specifying combinations of NR agonists and antagonists. Studies on proteomics and metabolomics in serum and plasma will provide new information on on-going systems changes induced by pro-anakoinotic therapy approaches.

A randomized trial introducing in the experimental arm metronomic low-dose chemotherapy, a selective inhibitor of the enzyme steroid-17*α*-hydroxylase (CYP17A1), which catalysis steps in the testosterone and estrogen biosynthesis, and dual transcriptional modulation with glucocorticoid and pioglitazone is ongoing in castration-resistant prostate cancer. In a second on-going trial, pioglitazone and all-trans retinoic acid are combined with azacitidine in the experimental arm for treating refractory acute myelocytic leukemia.

## Figures and Tables

**Table 1:** Explanation of communication-associated terms.

Communication-Associated Terms	Explanation
Anakoinosis	Anakoinosis is a novel paradigm for cancer treatment based on a key role for communicative reprogramming of tumor systems. Building on a systems biology approach to cancer, anakoinosis utilizes a range of non-cancer and cancer drugs in combination to treat advanced tumor disease, such as pioglitazone. In contrast to standard therapies, anakoinosis protocols are characterized by low toxicity and a good safety profile, with encouraging responses in a number of clinical trials to date. The use of drug repurposing, that is the use of non-cancer drugs as cancer treatments, is especially a notable feature of this approach.
Pro-anakoinotic therapeutic tools (examples)	Transcriptional modulators, nuclear receptor agonists and antagonists, metronomic low-dose chemotherapy, cyclooxygenase-2 inhibitors, IMiDs, arsenic trioxide, liposomal encapsulated small oligonucleotide encoding small activating RNAs, etc.
Metronomic tumor therapy	Metronomic tumor therapy may be defined as the frequent administration of (repurposed) drugs at doses significantly below the maximum tolerated dose with no prolonged drug-free breaks, or as the minimum biologically effective dose of an agent given as a continuous dosing regimen with no prolonged drug-free breaks that still leads to anti-tumor activity.
Rationalizations	Describe the physical organization of tumor-associated normative notions (e.g. hallmarks of cancer); are to some degree histology- and genotype-independent; may be re-directed and reorganized by anakoinosis.
Metabolism of evolution	The sum of extrinsically, i.e., therapeutically, and intrinsically inducible evolutionary processes within the tumor environment (tumor stroma, hosting organ, distant organ sites).
Modularity	Modularity describes the degree and specificity to which systems’ objects, i.e. cells, pathways, molecules, therapeutic targets etc. may be communicatively rededicated by anakoinosis.
Validity and denotation	Validity of systems objects, functions and hubs: Availability on demand at distinct systems stages; denotation: Current functional impact at a distinct systems stage, e.g. of potentially tumor-promoting pathways. In the bio-world, presence and functioning of an object (e.g., an enzyme), respectively.

**Table 2:** Glitazones including treatment schedules in metastatic cancer or hematologic neoplasia.

	Glitazones in Refractory Tumors or Hematologic Neoplasia					
Neoplasia	No pts	Chemotherapy (* = Metronomic)	Transcriptional Modulators	Small Molecule	Best Response	Reference
**Sarcomas**						
Liposarcomas, intermediate to high-grade (case reports)	-	-	Troglitazone	-	Histological and biochemical differentiation	[45]
Liposarcoma	3	Trofosfamide *	Troglitazone	-	Lineage-appropriate differentiation can be induced pharmacologically in a human solid tumor.	[46]
Liposarcoma (Phase II study)	12	-	Rosiglitazone	-	Rosiglitazone is not effective as an antitumoral drug in the treatment of liposarcomas	[47]
Kaposi sarcoma, refractory	1	Trofosfamide *	Pioglitazone	COX-2 inhibitor	Partial remission	[48]
(Hem-)angiosarcomas	12	Trofosfamide *	Pioglitazone	COX-2 inhibitor	Continuos complete remission	[49]
**Breast cancer**						
Refractory breast cancer (Phase II study)	22	-	Troglitazone	-	No significant effect	[50]
**Melanoma**						
Melanoma III (versus DTIC), phase II Clinical Trials.gov:NCT01614301	6	Trofosfamide *	Pioglitazone	Temsirolimus COX-2 inhibitor	Partial remission, Resolution of cachexia	[51]
Melanoma (randomized)						
Melanoma II Arm M	35	Trofosfamide *	Pioglitazone	-	Stable disease	[52]
Arm A/M	32	Trofosfamide *	Pioglitazone	COX-2 inhibitor	Partial remission	
**Hepatocellular carcinoma**						
Hepatocellular carcinoma	38	Capecitabine *	Pioglitazone	COX-2 inhibitor	Partial remission	[4]
**Cholangiocellular carcinoma**						
Cholangiocellular carcinoma	21	Trofosfamide *	Pioglitazone	COX-2 inhibitor	Partial remission	[18]
**Colorectal cancer**						
Chemotherapy-resistant metastatic colorectal cancer (phase II study)	25	-	Troglitazone	-	Not active for the treatment of metastatic colorectal cancer	[53]
**Renal clear cell carcinoma** (historic comparison)						
Renal clear cell carcinoma, relapsed	18	Capecitabine *	Pioglitazone	COX-2 inhibitor	Partial remission	[54]
Renal clear cell carinoma, relapsed	33	Capecitabine *	Pioglitazone Interferon-alpha	COX-2 inhibitor	Continuous complete remission	[5]
**Prostate cancer**						
Prostate cancer	41	-	Troglitazone	-	Lengthened stabilisation of prostate-specific antigen	[55]
Castration-resistant prostate cancer	61	Treosulfan *	Pioglitazone, Dexamethasone	COX-2 inhibitor Imatinib	Long-term tumor control at minimal disease	[56]
Castration-resistant prostate cancer	36	Capecitabine *	Pioglitazone, Dexamethasone	COX-2 inhibitor	Long-term tumor control	[57,58]
**Prostate carcinoma** (randomized)						
Rising serum prostate-specific antigen level after radical prostatectomy and/or radiation therapy	106	-	Rosiglitazone *Versus* Placebo		Rosiglitazone did not increase PSA doubling time or prolong the time to disease progression	[59]
**Gastric cancer** (randomized)						
Gastric cancer Arm A/M	21	Capecitabine *	Pioglitazone	COX-2 inhibitor	Partial remission	[60]
Arm M	21	Capecitabine *			Pioglitazone no impact	
**Glioblastoma**						
Glioblastoma, refractory	14	Capecitabine *	Pioglitazone	COX-2 inhibitor	Disease stabilization	[61]
**Multiple myeloma**						
Multiple myeloma, third-line Clinicaltrials.gov, NCT001010243	6	Treosulfan *	Pioglitazone, Dexamethasone	Lenalidomide	Complete remission	[62]
**Langerhans cell histiocytosis**						
Langerhans cell histiocytosis, refractory	2 + 7	Trofosfamide *	Pioglitazone Dexamethasone	COX-2 inhibitor	Continuous complete remission	[13,63,64]
**Hodgkin‘s lymphoma**						
Hodgkin lymphoma, refractory	3	Treosulfan *	Pioglitazone, Dexamethasone	COX-2 inhibitor Everolimus	Continuous complete remission	[65]
**Chronic myelocytic leukemia**						
Chronic myelocytic leukemia without moleclar CR	24	-	Pioglitazone	Imatinib	Molecular complete remission (54%)	[66]
Acute myelocytic leukemia						
Acute myelocytic leukemia Refractory (on-going trial)	5 + 7	Azacitidine	Pioglitazone All-trans retinoic acid		Molecular complete remission Myelodysplastic synrome with phagocytically active blasts	[67,68]
